# Use of anionic denaturing detergents to purify insoluble proteins after overexpression

**DOI:** 10.1186/1472-6750-12-95

**Published:** 2012-12-11

**Authors:** Benjamin Schlager, Anna Straessle, Ernst Hafen

**Affiliations:** 1 Institute for Molecular Systems Biology, ETH Zurich, Wolfgang Pauli-Strasse 16, Zurich, 8093, Switzerland

**Keywords:** Inclusion Bodies, Sodiumdodecylsulphate (SDS), N-lauroylsarcosine sodium salt (Sarkosyl), Immobilized Metal Ion Affinity Chromatography (IMAC)

## Abstract

**Background:**

Many proteins form insoluble protein aggregates, called “inclusion bodies”, when overexpressed in *E. coli*. This is the biggest obstacle in biotechnology. Ever since the reversible denaturation of proteins by chaotropic agents such as urea or guanidinium hydrochloride had been shown, these compounds were predominantly used to dissolve inclusion bodies. Other denaturants exist but have received much less attention in protein purification. While the anionic, denaturing detergent sodiumdodecylsulphate (SDS) is used extensively in analytical SDS-PAGE, it has rarely been used in preparative purification.

**Results:**

Here we present a simple and versatile method to purify insoluble, hexahistidine-tagged proteins under denaturing conditions. It is based on dissolution of overexpressing bacterial cells in a buffer containing sodiumdodecylsulfate (SDS) and whole-lysate denaturation of proteins. The excess of detergent is removed by cooling and centrifugation prior to affinity purification. Host- and overexpressed proteins do not co-precipitate with SDS and the residual concentration of detergent is compatible with affinity purification on Ni/NTA resin. We show that SDS can be replaced with another ionic detergent, Sarkosyl, during purification. Key advantages over denaturing purification in urea or guanidinium are speed, ease of use, low cost of denaturant and the compatibility of buffers with automated FPLC.

**Conclusion:**

Ionic, denaturing detergents are useful in breaking the solubility barrier, a major obstacle in biotechnology. The method we present yields detergent-denatured protein. Methods to refold proteins from a detergent denatured state are known and therefore we propose that the procedure presented herein will be of general application in biotechnology.

## Background

The purification of natively folded protein from heterologous expression systems is cumbersome and in some cases impossible because of the formation of insoluble protein aggregates called inclusion bodies [1]. Factorial screens of expression conditions or refolding assays can yield soluble proteins in some cases and can increase yield in most cases but many proteins remain resistant [2]. Affinity purification under denaturing conditions followed by renaturation can yield natively folded protein and is a viable alternative.

Anfinsen first demonstrated the reversible denaturation of proteins in solutions of urea in the 1960ies [3]. It has since been the method of choice to denature native proteins and also aberrant protein aggregates. The denaturation of proteins by urea yields protein in a random coil state, i.e. no secondary structure elements are favoured over any other conformation [4]. The interactions of SDS with proteins have been intensively studied since the 1940ies [5-8]. Denaturation of polypeptides by SDS is a multi-step process that starts with the interaction of the negatively charged sulphate group with oppositely charged, basic, amino acid side chains [9]. The hydrophobic tails of SDS molecules then become buried in the hydrophobic core of proteins and start to disrupt the native structure [10-12]. Finally a large fraction of the polypeptide chain, independent of its conformation in the native state, adopts an alpha-helical conformation and is surrounded by a micelle of SDS molecules [9,13]. The length of this mixed protein-detergent micelle is roughly proportional to the molecular weight of the polypeptide [6].

Thus the denaturation of polypeptides by urea and SDS are different at the mechanistic level and yield two different results: a random-coil structure in urea and a largely alpha-helical conformation in SDS [4,6].

Hexahistidine-tagged proteins can be purified under denaturing conditions using chaotropic concentrations of urea or guanidinium hydrochloride [14]. The high-affinity binding of the hexahistidine tag to Ni/NTA resin is based on the co-operative co-ordination of a nickel cation by two histidine side chains [14]. It is independent of the peptide conformation but requires close physical proximity (reviewed in [14]).

We wondered whether the histidine side chains of an SDS denatured protein, which are buried inside a mixed detegent-protein micelle, would be accessible for binding to Ni/NTA and whether thus detergent denatured proteins could be purfied by immobilized metal ion affinity chromatography (IMAC).

## Methods

### Reagents and chemicals

Phusion Polymerase was from New England Biolabs (Ipswich, USA). Cloning vector pET151/D-TOPO and BL21 cells were from Life Technologies (Zug, Switzerland). All chemicals used in this study were laboratory-grade. IPTG and SDS were from Carl Roth GmBH (Karlsruhe, Germany). DTT, imidazole and carbenicillin were from Applichem (Darmstadt, Germany). N-Lauroylsarkosine (Sarkosyl) was from Sigma-Aldrich (Steinheim, Germany) and NHS-activated Sepharose was from GE Healthcare Biosciences (Uppsala, Sweden). Purification was done on 5 ml HisTrap columns from GE Healthcare Biosciences (Uppsala, Sweden). Precast SDS-PAGE gels were from LucernaChem (Luzern, Switzerland) and were stained with Coomassie Brilliant Blue as described elsewhere [15].

### Plasmid construction

Expression constructs and their inserts are detailed in Table 1. cDNA was made from 0–6 hour old *Drosophila melanogaster* embryos or from *Drosophila* KC cells. Expression constructs were generated by blunt-end PCR amplification from cDNA using Phusion Polymerase. Amplified PCR fragments were cloned into pET151/D-TOPO to generate expression constructs that express a 6xHis-V5-TEV tagged fusion protein. All inserts were confirmed to be in-frame and full length by sequencing.

**Table 1:** Expression constructs and details of inserts

**Plasmid number**	**Insert Protein Domain**	**Flybase Protein ID and AA range**	**Uniprot ID**	**MW of fusion protein in kDa**
**165**	chico PH	chico-PA	Q9XTN2	15.6
		8-107		
**166**	chico PTB	chico-PA	Q9XTN2	16.6
		122-235		
**168**	Pi3K92E catalytic	Pi3K92E-PA	P91634	45.7
		726-1088		
**171**	PDK1 PH-like	Pdk1-PA	Q9W0V1	14.3
		593-680		
**172**	Akt1 catalytic	Akt1-PA	Q8INB9	40.3
		189-509		
**173**	rictor REM	rictor-PA	Q9VWJ6	12.1
		827-896		
**174**	Tor kinase catalytic	Tor-PA	Q9VK45	36.3
		2074-2352		
**175**	S6K catalytic	S6k-PA	P91656	39.2
		92-402		
**176**	TSC2 Tuberin	gig-PA	Q9VW83	40.5
		561-883		
**177**	TSC2 DUF 3384	gig-PA	Q9VW83	54.3
		37-473		
**178**	raptor WD	raptor-PA	Q9W437	47.9
		1210-1624		
**179**	InR Y-kinase	InR-PA	P09208	36.7
		1363-1625		
**180**	Akt1 PH-like	Akt1-PA	Q8INB9	16.0
		26-129		
**181**	foxo winged helix	foxo-PB	Q95V55	13.1
		95-175		
**182**	Tor kinase DUF 3385	Tor-PA	Q9VK45	22.8
		830-998		
**183**	Pi3K21B N-term SH2	Pi3K21B-PA	O18683	16.6
		24-133		
**184**	Pi3K92E accessory	Pi3K92E-PA	P91634	24.6
		552-723		

All constructs are based on pET151/D-TOPO. This vector contains a 6xHis-V5 Epitope - TEV cleavage sequence tag of approximately 4 kDa. The molecular weights given are those of the entire fusion protein, i.e. containing the tag.

### Overexpression in *E. coli*

Expression constructs were heat-shock transformed ino BL 21 (DE3) STAR cells, plated on LB plates containing carbenicillin (50 μg/ml) and incubated o/n at 37°C. The next day several colonies were collected and used to inoculate 300 ml cultures of LB medium containing carbenicillin. The cultures were grown at 37°C until the OD 600 was 0.5 and then induced by addition of IPTG to a final concentration of 0.5 mM. Cultures were then grown over night at 30°C.

### Buffered solutions

**PCL** (lysis buffer) contained 8 mM Na2HPO4, 286 mM NaCl, 1.4 mM KH2PO4, 2.6 mM KCl and 1% SDS (w/v) at pH 7.4. **PCW** (wash and equilibration buffer) contained 8 mM Na2HPO4, 286 mM NaCl, 1.4 mM KH2PO4, 2.6 mM KCl and 0.1% Sarkosyl (w/v) at pH 7.4. **PCE** (elution buffer) contained 8 mM Na2HPO4, 286 mM NaCl, 1.4 mM KH2PO4, 2.6 mM KCl, 500 mM imidazole and 0.1% Sarkosyl (w/v) at pH 7.4.

### Purification

The cultures were harvested in GS3 rotor tubes by centrifugation at 4°C for 12 minutes at 6000 rpm. The pellet was resuspended in 30 ml PCL buffer, supplemented with DTT to 1 mM, and sonicated with a Bandelin Sonoplus HD2070 sonicator, set to 80% cycle and 40% power using a MS73 probe-tip (Bandelin electronic, Berlin, Germany). Samples were sonicated at room temperature twice for 2 minutes each. The lysates were transferred to SS34 tubes and placed in an ice-water mixture and incubated for 30 minutes. The chilled lysates were then centrifuged in a SS34 rotor at 13 krpm for 20 minutes at 4°C. The cleared supernatant was poured off and filtered through a 0.45 μm syringe filter before applying it to affinity purification. Ni/NTA affinity purification was performed on an AKTA Xpress FPLC system using 5 ml HisTrap HP columns and standard purification templates (GE Healthcare Biosciences Uppsala, Sweden). Columns were equilibrated with PCW buffer, the lysate loaded and the columns washed until the absorption of post-column flowthrough returned to base levels. Bound proteins were eluted with a 100 ml linear gradient of buffers PCL and PCE, from 0 to 50% buffer PCE, i.e. from 0 to 250 mM imidazole in 20 column volumes. Weakly bound contaminating proteins typically eluted at 40 mM imidazole, the peak maximum of hexahistidine tagged proteins was between 80 and 150 mM imidazole.

**Figure 1:**
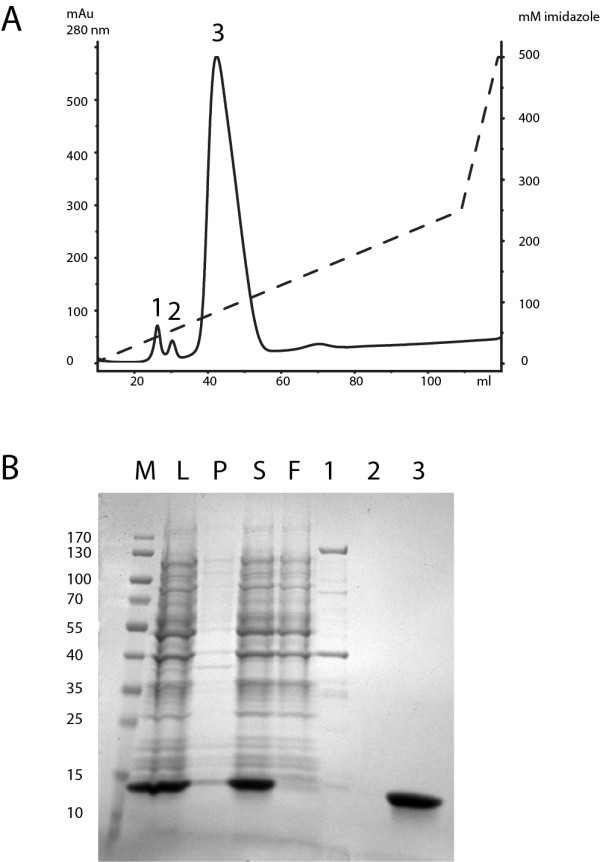
**IMAC purification of an SDS-denatured hexahistitine-tagged protein from inclusion bodies. A** Elution profile of a representative protein (173, see Table 1 for details). The x-axis shows the volume in ml during elution of a 5 ml-HisTrap column. The left y-axis shows arbitrary absorbance units at 280 nm, the right y axis shows the concentration of imidazole during elution. Absorbance is shown in a solid line, imidazole concentration as a dashed-line. Three distinct peaks are seen. Samples of these peaks were loaded onto the gel shown in Figure 1B. **B** Coomassie stained SDS-PAGE gel showing samples of the purification procedure of construct 173. Samples are labeled as follows: **M:** Molecular weight marker. **L:** Lysate in PCL buffer after sonication. **S:** Supernatant after cooling out SDS and centrifugation. **F**: Combined flowthrough and wash after binding to Ni/NTA Sepharose. **1,2,3** Samples of the three peaks seen in the elution profile shown in Figure 1A.

**Figure 2:**
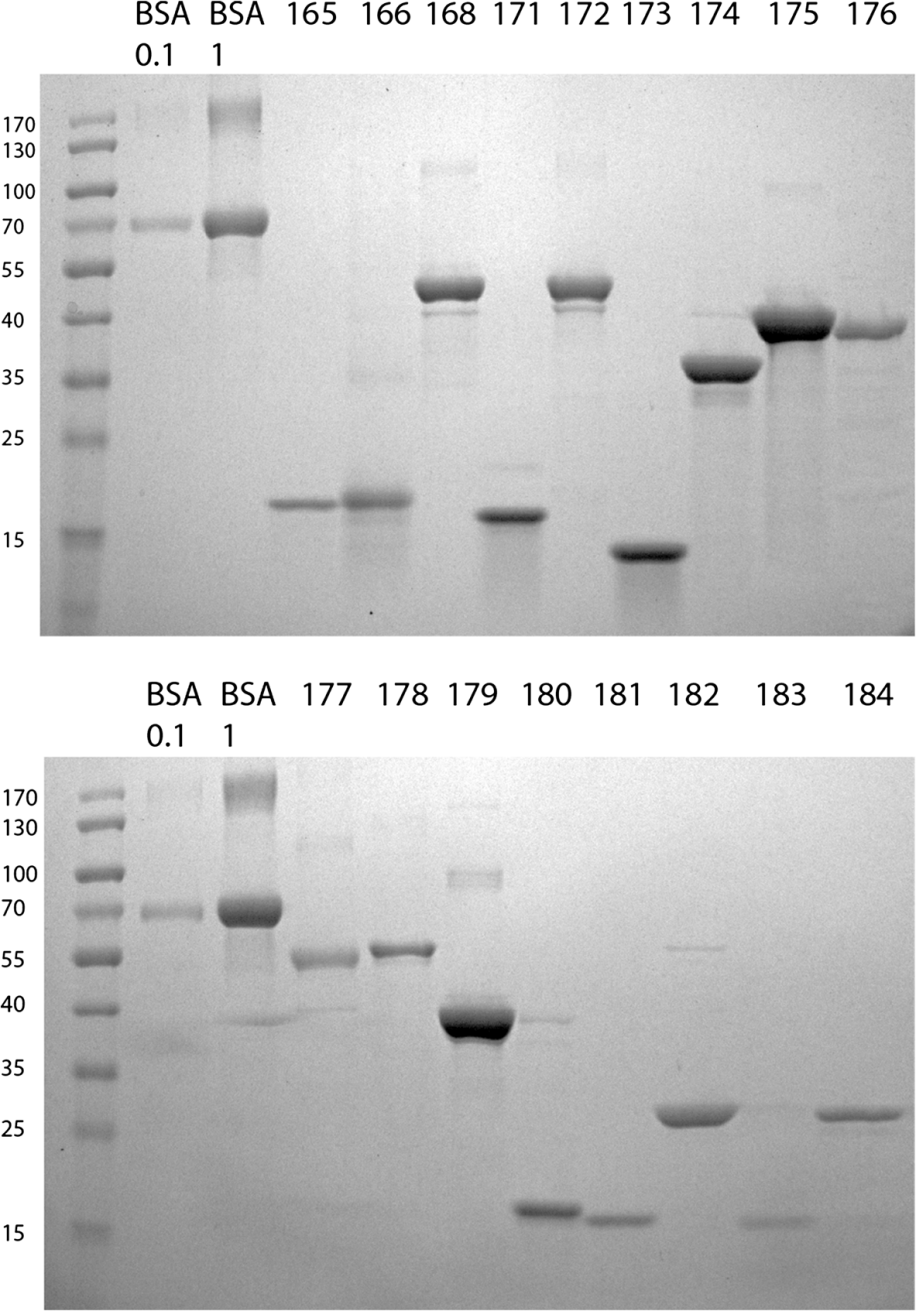
**IMAC of SDS-denatured proteins is a generic method for protein purification.** Coomassie stained SDS-PAGE gels that show the purity of seventeen purified proteins. Lanes are labelled as follows: **BSA 0.1** and **BSA 1:** Samples were prepared from BSA stock solutions containing 0.1 mg/ml and 1 mg/ml BSA respectively by adding 40 μl 5X SDS Sample buffer to 40 μl of stock solution. 15 μl each were loaded onto the gel - containing 0.75 and 7.5 μg BSA total. Numbers **165**–**184** indicate the expression construct and protein purified. See Table 1 for details of expression constructs. Samples were prepared and loaded as for the BSA control samples.

Samples were taken at various points (see legend Figure 1) and loaded on SDS-PAGE gels according to the total volume of the fraction to make samples comparable. SDS PAGE was performed on 12% 17-well gels according to the manufacturers recommendations. See Figure 1 for a Coomassie stained SDS-PAGE gel of one representative sample (Construct 173). See Figure 2 for samples of the final eluted fractions for all 17 proteins.

## Results

Figures 1 and 2 present the purification of seventeen different fusion proteins of sizes between 13 and 54 kDa, detailed in Table 1. These proteins were previously shown to be insoluble after lysis of induced cells in standard native lysis buffers (PBS containing 0.5% Triton X-100 or 0.3% Sarkosyl, data not shown). They are domains of proteins involved in the *Drosophila* Insulin and Tor Kinase signaling network and represent various non-homologous structures [16]. The purification of one of these protein domains, the REM domain of rictor, encoded by construct 173, is shown in detail in Figure 1. For the other sixteen proteins only the final, purified protein, is shown in Figure 2.

The hexahistidine-tagged proteins were overexpressed in *E. coli* using standard procedures (see Materials and Methods). After harvest the cells were resuspended and sonicated in a buffer containing high concentrations of SDS (34 mM, 1% w/v). Proteins that aggregated in inclusion bodies were rapidly dissolved by sonication (see Figure 1, lane “L”). The lysates were then cooled to precipitate free SDS. The SDS pellet contained only small amounts of protein (see Figure 1, lane “P”). After centrifugation the supernatant contained both host proteins and overexpressed target protein (see Figure 1, lane “S”, compare to “L”). Thus proteins do not co-precipitate with SDS when the lysate is chilled. The lysates were then subjected to purification as detailed in Materials and Methods. The combined flow-through and wash fraction contained only minimal amounts of unbound fusion protein (see Figure 1, lane “F”). The elution profile shows three consecutive peaks eluting at ±20, 40 and 90 mM imidazole (Figure 1A). Table 2 summarizes the purification procedure.

**Table 2:** Summary of the purification procedure

**Step**	**Description**	**Purpose**	**Comments**
1	Growth, induction and harvest of induced cells	Overexpression of target protein in *E. coli*	As described elsewhere [23]
2	Resuspension in 1% SDS in PBS	Lysis of cells	Works fine in 1/20 to 1/10 of the original culture volume. Other buffers also work (50 mM Tris/Cl pH 7.0, 100 mM NaCl and 1% SDS)
3	Sonication	Solubilisation of proteins from inclusion bodies	Until solution turns clear. Often faster than 2 minutes.
4	Incubation on ice for 30 minutes	Precipitation of SDS	Precipitation of SDS apparent after 5 minutes. 1 h incubations on ice possible.
5	Centrifugation	Removal of precipitated SDS	SS34 rotor, 13 krpm, 20 minutes, 4°C
6	Ni/NTA affinity purification	Capture and washing of hexahistidine tagged target protein	See M&M for buffer compositions and details.
7	Elution in 0.1% Sarkosyl	Elution in a dialyzable detergent that is compatible with refolding [24]	Other detergents could be used but were not tested.

Key steps in the procedure are described and their purpose briefly explained. The comments are based on the experiments described herein and additional purifications using the same principle but with slightly different protocols (altered buffers, volumes and incubation time on ice, data not shown).

## Discussion

We wanted to test whether hexahistidine-tagged proteins can be IMAC-purified after SDS denaturation. In our first experiments we included 34 mM (1% w/v) of SDS in all buffers, i.e. in the lysis, wash and elution buffers commonly used for affinity purification. Cell pellets were rapidly lysed by sonication and binding to Ni/NTA resin occurred, however most of the hexahistidine-fusion protein was found in the flow-through, indicating poor binding (data not shown). Upon searching the literature we realized that there was no primary literature concerning the binding of hexahistidine-tagged proteins to Ni/NTA in solutions containing SDS. One manufacturer of Ni/NTA resins publishes a handbook that indicates that no more than 0.3% SDS should be included in the buffers [17] but these are empirical values that are not based on published experiments (QIAGEN AG, Basel Switzerland, pers. comm.).

In 1988 Suzuki and Terada published that SDS can be selectively removed from solutions containing BSA by cooling [8]. We hypothesized that the same principle might be applied to whole cell lysates and that it should yield a solution with lower concentrations of SDS that would be compatible with high affinity binding to Ni/NTA. We modified the buffer compositions such that only the buffer used for initial lysis contained high concentrations of SDS (34 mM, 1% w/v) and included a low concentration of Sarkosyl (3 mM, 0.1% w/v) in the wash and elution buffers. After lysis by sonication the lysate was cooled in an ice/water bath for 20 minutes and precipitated SDS removed by centrifugation. The residual concentration of SDS was compatible with high affinity binding to NI/NTA. We applied this purification protocol to seventeen different proteins (detailed in Results) and consistently achieved high-affinity binding and elution profiles that indicated sensitivity to imidazole concentration, i.e. contaminating proteins eluted at lower imidazole concentrations than the tagged target protein.

We would like to mention that we did not specifically test whether the dodecylsulphate anion (DS) of SDS precipitates as the sodium salt (SDS) or with another monovalent cation such as potassium (KDS), which is also present in the buffer [8].

Detergents display complex phase diagrams in aqueous solutions [7]. Many detergents separate into a distinct phase when temperature or salt concentrations change. A landmark paper showed that integral membrane proteins remain in the detergent phase after temperature induced phase separation of Triton X-114 and that therefore phase separation can be used as a tool in protein purification [18]. Nowadays phase separation of detergents, sometimes called “cloud point extraction”, is frequently used to purify membrane proteins [19].

We would like to point out that our method is similar to cloud point extraction only in as far as the detergent is used to initially solubilize the proteins of interest, in our case from inclusion bodies, and that phase separation is induced experimentally. However, it critically differs from cloud point extraction in that the proteins do not co-partition into the detergent phase after phase separation. Instead the surplus of unbound detergent is removed from the solution to allow subsequent affinity purification.

Curiously as early as in 1944 it was shown that SDS can be selectively removed from solutions containing proteins by cold precipitation with barium chloride [5]. Suzuki and Terada showed that SDS can be removed from solutions containing BSA by cooling [8], however, we are not aware of a publication that would combine this principle with the dissolution and denaturation of inclusion bodies and subsequent IMAC.

The buffer components used in this method are compatible with automated chromatography and allow high throughput purification of target proteins on a suitable purification platform. One key advantage over purification in urea or guanidinium is that SDS can be used at comparably low concentrations (34 mM versus 6–8 M) and does not tend to crystallize in valves and pumps of FPLC chromatographs [20].

Finally, we wish to add that protocols for the refolding of proteins from a detergent denatured state are known. One relies on replacing SDS with urea and subsequent removal of urea [21]. The other is based on cyclodextrine mediated stripping of detergent molecules from the protein [22]. We therefore think our method can be of application in the purification and refolding of recalcitrant proteins.

## Conclusion

Ionic, denaturing detergents are useful reagents in the solubilization and purification of proteins from inclusion bodies and can be used to replace the more commonly used reagent urea.

## Competing interests

The authors declare no competing financial interests.

## Authors’ contributions

AS cloned the expression constructs. BS planned and conducted all experiments and prepared the manuscript. All authors read and approved the final manuscript.
